# Structure of the Epiphyte Community in a Tropical Montane Forest in SW China

**DOI:** 10.1371/journal.pone.0122210

**Published:** 2015-04-09

**Authors:** Mingxu Zhao, Nalaka Geekiyanage, Jianchu Xu, Myo Myo Khin, Dian Ridwan Nurdiana, Ekananda Paudel, Rhett Daniel Harrison

**Affiliations:** 1 Key Laboratory for Plant Diversity and Biogeography of East Asia, Kunming Institute of Botany, Chinese Academy of Sciences, Kunming, Yunnan, China; 2 University of Chinese Academy of Sciences, Beijing, China; 3 Laboratory of Tropical Forest Resources and Environments, Division of Forest and Biomaterial Science, Graduate School of Agriculture, Kyoto University, Kitashirakawa Oiwake, Kyoto Prefecture, Japan; 4 Department of Plant Sciences, Faculty of Agriculture, Rajarata University of Sri Lanka, Anuradhapura, Sri Lanka; 5 World Agroforestry Centre, East Asia Office, Kunming, Yunnan, China; 6 Wildlife Conservation Society, Yangon, Myanmar; 7 Cibodas Botanic Garden, Indonesian Institute of Sciences, Cianjur, West Java, Indonesia; DOE Pacific Northwest National Laboratory, UNITED STATES

## Abstract

Vascular epiphytes are an understudied and particularly important component of tropical forest ecosystems. However, owing to the difficulties of access, little is known about the properties of epiphyte-host tree communities and the factors structuring them, especially in Asia. We investigated factors structuring the vascular epiphyte-host community and its network properties in a tropical montane forest in Xishuangbanna, SW China. Vascular epiphytes were surveyed in six plots located in mature forests. Six host and four micro-site environmental factors were investigated. Epiphyte diversity was strongly correlated with host size (DBH, diameter at breast height), while within hosts the highest epiphyte diversity was in the middle canopy and epiphyte diversity was significantly higher in sites with canopy soil or a moss mat than on bare bark. DBH, elevation and stem height explained 22% of the total variation in the epiphyte species assemblage among hosts, and DBH was the most important factor which alone explained 6% of the variation. Within hosts, 51% of the variation in epiphyte assemblage composition was explained by canopy position and substrate, and the most important single factor was substrate which accounted for 16% of the variation. Analysis of network properties indicated that the epiphyte host community was highly nested, with a low level of epiphyte specialization, and an almost even interaction strength between epiphytes and host trees. Together, these results indicate that large trees harbor a substantial proportion of the epiphyte community in this forest.

## Introduction

### Background

Epiphytes are plants that attach themselves to and grow on other plants but, in contrast to parasitic plants, epiphytes do not extract nutrients from the host's vascular system. The host provides only a place to grow. Epiphytes occur from the forest understory to the periphery of tree crowns [1], and a recent study collated a global list of 27,614 species of vascular epiphytes, which is about 9% of all vascular plant species [2]. At local scales, the structure of epiphyte community is influenced by a wide spectrum of biotic and abiotic factors, and its structural properties can be examined in a way analogous to a bipartite food web or a pollination network [3]. However, comprehensive study of the properties and factors structuring epiphyte communities is rare and lags far behind that of other components of plant communities, such as trees, shrubs or ground cover vegetation [4].

### Factors structuring epiphyte community

Many studies have shown that host size is an important factor in determining the diversity of epiphytes [5–7]. However, host size is a complex factor that integrates several ecological processes affecting epiphytes. A larger host can be related to a longer period of exposure to epiphyte seed rain, a larger surface area for colonization and an elevated epiphytic habitat with a greater range in abiotic conditions, such as light and humidity [8]. For these reasons, host size is also expected to be an important factor in structuring epiphyte communities. However, although its probable effects have been widely discussed, few studies have examined the effect of host size in determining variation in epiphyte species assemblages (but see Harrison et al. [9]). Moreover, contrary to expectations, Zotz [10] found host size explained less than 10% of the total variation in epiphyte assemblages on BCI in Panama. An improved understanding of the role of host size in structuring epiphyte communities, based on empirical study, is required.

Bark texture is another important host trait in the process of epiphyte colonization. Benzing [1], working in the Neotropics, demonstrated that factors such as moisture retention, chemical composition and bark morphology, could be important in the establishment and development of some epiphytic species. A study compared the stem characteristic of angiosperm hosts and tree ferns suggested that bark characteristics may favor the germination and establishment of certain species [11]. A recent study specialized on bark functional ecology found increasing bark thickness significantly increased water storage, which was vital to the survival of epiphytes [12]. Bark texture was also proved to be an important factor in determining epiphyte-host network structure [3]. Nevertheless, the extent to which variation in the epiphyte community assemblage can be explained by bark characteristics is still unclear.

Studies on the distribution of vascular epiphytes have often revealed a hump-shaped relationship between species richness and elevation [13, 14]. Thus, elevation is also expected to be an important factor in structuring the epiphyte community. Within hosts, epiphyte species richness is highest in the mid-canopy [10], which may be partly explained by the relative large surface area, the accumulation of substrate in the main fork site [15], and the intermediate levels of light and moisture [16]. Most epiphytes have tiny seeds (or spores) that are dispersed by wind [1]. In an attempt to model epiphyte seed rain, Shaw et al. [17] conducted a smoke dispersion experiment in forest and found that the smoke always formed horizontal layers in different forest strata. This suggests that the height of the main fork may be important in determining the epiphyte seed rain it is exposed to, and hence that stem height may be important in structuring epiphyte community composition.

Light is an essential factor for all green plants and low or, conversely, excessive photon flux may affect the species composition of the epiphyte community [1]. We adapted the five point scale of crown illumination index from a former study to assess the light environment of the host crown and the light conditions of each epiphyte [18]. Another factor that is commonly assessed in studies on epiphytes is the height above ground [10, 19], which may be a proxy for light and humidity [16, 20]. Water and nutrients available to epiphytes are likely to be directly linked to the nature of the rooting substrate [1, 16]. We assessed epiphyte rooting substrate visibly to investigate whether this was an important factor in determining epiphyte community structure.

### Epiphyte-host Network

Burns [8] borrowed the meta-community concept from biogeography, food webs and pollination studies as a means to describe the bipartite network of epiphytes on host trees. Using this approach the species distribution pattern, specialization and interaction evenness of epiphyte-host ecological network can also be explored. When applying the concept to an epiphyte-host community in New Zealand, he found the network was highly nested [8]. However, subsequently he found a compartmentalization pattern of epiphyte-host interactions in a temperate rainforest [21]. Others working in Mexico found a nested pattern [3]. These studies involved relatively species poor communities or only a subset of epiphyte species. Burns [8, 21] studied communities with only nineteen and nine vascular plant species, respectively, and Sáyago et al. [3] only studied bromeliads. Thus, there is a need to expand this approach to more diverse epiphyte-host communities.

We investigated the properties of the host-epiphyte community in an old growth forest in relation to the factors described above. Specifically, we addressed the following questions; (i) how is epiphyte species richness and abundance distributed with respect to host size (tree DBH and height), stem height, bark texture, elevation, light (host and within host levels), branch position, crown position and substrate; (ii) how is variation in the epiphyte community composition structured with respect to these factors, both among hosts and within hosts; and (iii) what are the properties of the epiphyte-host network?

## Methods

### Study site

**Fig 1:**
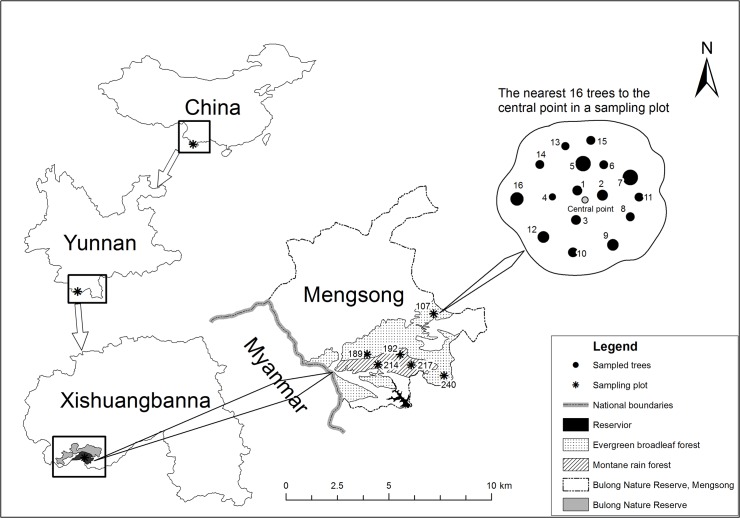
Location of Bulong Nature Reserve Mengsong. The map was drawn in ArcGIS 10.2, and the boundaries were digitalized from the official map, representative purposes only herein. The old growth forests were textured, while the areas left blank were mainly covered by secondary forest and open land.

Our study was conducted in a recently established nature reserve (Bulong Nature Reserve, Mengsong) in Xishuangbanna, SW China (Fig 1, outlines of tropical montane rain forest were adapted from [22], and modified in our field work). Annual mean temperature is 18°C at 1,600 m a.s.l. (above sea level). The annual rainfall ranges between 1,600–1,800 mm and 80% of the precipitation falls between May and October. The vegetation shows altitudinal zonation and the history of anthropogenic activities and micro-environment has resulted in a patchy distribution of flora. There are two forest types: a tropical evergreen broadleaf forest (main part) and a tropical montane rain forest [23]. We surveyed vascular epiphytes in both of these forest types.

### Data collection

Field work was conducted in November, 2012. Six previously established 1 ha (100 × 100 m) permanent sampling plots in an area of old growth forest were selected for the study. The permanent plots were stratified according to different vegetation types; three plots were situated in evergreen broadleaf forest and three in montane rain forest, although these types inter-grade at the site (Fig 1 and S1 Table).

In each 1 ha plot, a central point was marked using a GPS unit (Garmin-etrex20) (Fig 1). From this point we surveyed the 16 nearest trees (>10 cm DBH) for epiphytes. Yang [24] found that 73 trees were required for a total survey of the epiphyte community at Huanglian Mountain Nature Reserve, Yunnan (240 km NE of Mengsong). Pilot tests in Mengsong revealed that approximately 80% of trees (>10 cm DBH) hosted epiphytes. Thus, we elected to survey the nearest 16 trees.

We did not consider sampling epiphytes from a fixed area, but chose an equal number of trees in each site to standardize our sampling effort, for the simple reason that epiphytes are directly linked with trees rather than the forest floor. Sampling a fixed area would have resulted in variable sampling effort per plot, which may have complicated comparisons among sites. We examined environmental factors at two levels, host tree traits (homogeneous for the whole epiphyte community on a tree) and micro-site environmental factors (heterogeneous among epiphyte individuals or clusters of individuals). Host factors included, *host size* (*DBH* and *tree height*), *stem height*, *bark roughness*, *canopy illumination index* and *elevation*. Microsite factors included, *crown position*, *branch level*, *light condition* and *substrate*. The specific methods for the acquisition of the data on these two types of factor were as follows.

**Fig 2:**
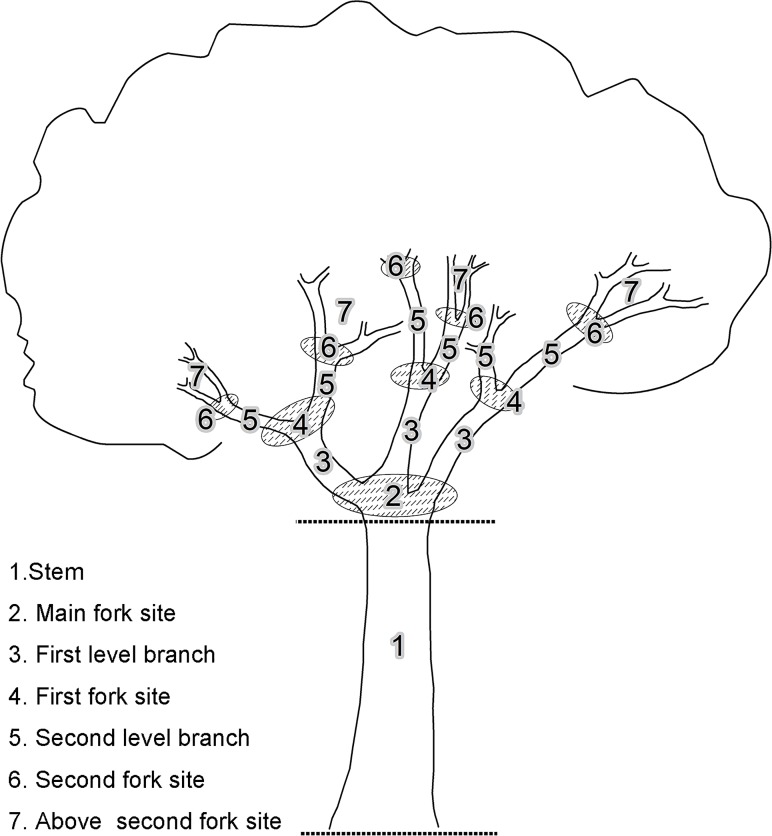
Coding for the within crown branch levels assignments.

We measured tree *DBH* at the height of 1.3 m above ground in accordance with the international practice. *Tree height* and *stem height* were measured using a 5 m pole, while trees or stems higher than the pole were measured by estimating the height relative to the pole with the observer placed at a distance of at least 20 m. *Bark roughness* as a semi-quantitative factor, was categorized into three categories according to the width of bark fissure from narrow to broad. Category 1: smooth bark and bark with narrow fissures (fissure width < 1 cm), such as *Albizia chinensis* (Osbeck) Merrill (Fabaceae), *Michelia floribunda* Finet & Gagnepain (Magnoliaceae); Category 2: moderately cracked bark (fissure width 1 cm ≤ 2 cm, including some young trees of Category 3 bark species, such as *Cinnamomum javanicum* Blume Bijdr. (Lauraceae) and *Castanopsis mekongensis* A. Camus (Fagaceae); Category 3: rough bark (fissure >2 cm), such as *Schima wallichii* (Candolle) Korthals (Theaceae) and *Betula alnoides* Buchanan-Hamilton ex D. Don (Betulaceae). Ornamentations of bark were omitted in the records, because of the rare occurrence of such tree species in this area. *Crown illumination index* (CII) is a user-assessed index on the amount of light reaching a tree crown. It consists of five categories from full overhead and side light (CII = 5), as for an emergent crown, to a crown in full understory shade (CII = 1) [18] (S2 Table). *Elevation* was measured using a GPS unit. We measured the *stem height* because different trees with similar total heights may have different stem heights, which thus affect the height of the main (or lower) fork site, one of the most important micro-habitats for epiphytes. Among microsite factors *crown position* was recorded as vertical distance from the epiphyte crown to the ground, measured or estimated using the 5 m pole. *Branch level* was recorded based on the relative position of the branches (Fig 2). We based our assessment of *branch level* on the Johansson analogous subdivision of hosts [25], which is used in many canopy studies [26, 27]. However, we used a slightly different scheme, in which seven divisions were used. Of zones 1–7, even number zones were forks, while odd number zones were the trunk and branches (Fig 2). Zone 7 included all branches and fork sties above zone 6. The *light condition* of each epiphyte site was estimated based on five categories similar to CII (S2 Table). The type of substrate was recorded as bare bark, canopy soil (here canopy soil refers to histosols developed from the accumulation and decomposition of bark debris and fallen leaves only), or mosses (moss covered mats). Although studies on canopy soil (both histosols and moss covered mats) now have drawn the attention of scientists [28–30], little is known about the differences in water and nutrients between non-moss covered canopy soil and moss covered mats, although this may be important for epiphytes. Moss covered mats are always located in more stable and humid parts of the crown, as bryophytes are highly sensitive to water, temperature, light and nutrient condition [31]. Hence, we ranked the three substrate types a semi-quantitative gradients as; bare bark = 1, canopy soil = 2 and moss mat = 3.

All epiphytes higher than approximately 5 cm were identified in the field with the aid of binoculars and a spotting scope [3, 32]. One of us (MZ) is an experienced field botanist who has conducted extensive surveys in Xishuangbanna. Specimens were collected using a telescopic pruning shears with maximum reach of 5 m and vouchers of each field species were deposited at the herbarium of the Xishuangbanna Tropical Botanical Garden, Menglun. Field names were checked against curated material and with reference to the *Flora of China* [33]. Plant names and families followed *Flora of China* online. Accidental epiphytes and parasitic plants were not included in our study and, to avoid any confusion of lianas and secondary epiphytes [2], we excluded secondary epiphytes from the hemiepiphyte category of Benzing’s [1] scheme I of epiphyte definitions, such as Aroids, Piperoids and climber *Ficus* spp. Only primary hemiepiphytes, such as some *Ficus* spp. and *Schefflera* spp. that initiate as epiphytic seedlings and secondarily root to the ground, were retained. Sanford’s [34] “stand” method was used to define epiphyte individuals: if a collection of individual stems or plants was spatially separated from another group of the same species, either by an area on the tree devoid of epiphytes or occupied by another species, then this was defined as an individual. When the same area was occupied by an intermingling of more than one species, one stand was counted for each species present.

### Data analysis

#### Factor Selection

To avoid problems of colinearity we reduced the number of environmental factors to a minimum set of uncorrelated factors. We investigated correlations among factors using Spearman’s rank correlation coefficient and used a threshold value of *ρ* < 0.5 (*p* > 0.01) for inclusion in the set of uncorrelated variables.

#### Non-metric Multidimensional Scaling Ordination

Data were organized into a site-by-species matrix, with cells filled with the numbers of individuals of each species. To visualize epiphyte community composition we used non-metric multidimensional scaling (NMDS), using Bray-Curtis dissimilarity, which was implemented in the package *vegan* [35] in *R* v3.0.0 [36]. We removed epiphytes of importance value index (IVI) less than 0.01. IVI was calculated as IVI = RF+RA, where RF (relative frequency) = frequency of a given species *i* / sum of the frequencies of all of the species and RA (relative abundance) = number of individuals of species *i* / total number of individuals. Such species provide little information on community assembly, but can substantially invalidate the goodness-of-fit (stress) of NMDS ordination and disturb the display of abundant species in a two dimension ordination plot (S1 Fig). The matrix was standardized, using Wisconsin double standardization, and we used the ‘*stressplot*’ function to evaluate the goodness-of-fit between fitted vectors and ordination values. To visualize the associations with the habitat factors, we plotted factor values as contours on the ordination biplot (function *ordisurf*).

#### Variance partition

Linear models were implemented to investigate the associations between epiphyte diversity (Shanon-Wiener index was chosen given to the large proportion of rare species) and environmental factors at the host tree level. Multivariate generalized linear models (function *manyglm*), were implemented in the package *mvabund* [37] in *R*, to investigate the relationship between environmental factors and epiphyte community composition at the host tree and branch level. We used a negative binomial error distribution, given our data is typical of site × species count data [37]. We used the plotting functions in *manyglm* to confirm our data conformed to a negative binomial distribution. *manyglm* models the univariate response of each species in the site × species matrix and sums across the univariate responses to estimate the multivariate response. Probabilities are assigned by comparison against a null distribution, which was based on 1,000 randomizations. Habitat factors were standardized to a mean of zero and a standard deviation of one. For the multivariate model, our response matrix was the quantitative site × species matrix and the independent variables were the selected independent factors and all two-way interactions.

We started with the maximal model and simplified models manually through removal of non-significant terms, respecting the principle of marginality, and used AIC values to determine the model performance. We also examined the role of the position of the independent variables in the model. Altogether, 15 models were constructed based on macro-site environmental factors. Additionally, to examine the performance of each model, an intercept only model (null model) was constructed and model performance was assessed as the proportional improvement in the deviance explained by the target model compared to the null model. To investigate the effects of micro-site factors in structuring the epiphyte community, we used a second multivariate GLM. We treated each of the seven tree zones (branch level zones) as a separate site and again populated the matrix using the numbers of epiphyte individuals. The micro-habitat conditions of each zone were assessed from the epiphyte level data. If conditions within a branch level zone of a host varied among any of the micro-habitat factors, the average condition for the zone was calculated. We compared models as described above. A total of seven models for micro-site environmental factors were constructed.

#### Analysis of network properties

The comprehensive study of ecological network indices proposed by Dormann et al. [38] and the *R* package *bipartite* they developed was used to calculate all of indices to describe the epiphyte-host network, although it was based on the work of pollination studies [38, 39]. Another *R* package *FALCON* was available to assist the judgment of nested structure with a full list of effective null models developed in the former studies [40]. We assessed the following indicies:

- *Nestedness temperature* (*T* = 0–100°, 0 for maximum nestedness): [41].
- NODF (Nestedness metric based on Overlap and Decreasing Fill) [42]: NODF varies from 0 (no nestedness) to 100 (perfect nestedness). N_rows_ and N_columns_ are a sum of the nestedness introduced by rows (host) and by columns (epiphyte), representing the independent contributions of hosts and epiphyte species to NODF (total nestedness), respectively.
- Discrepancy score[43]: The mismatch number of observed matrix compared with the maximally nested packed matrix (with same row sums), the *p* value is accessed against five types null models, which preserved some features of observed matrix [40], and each type null model was randomized at least of 1,000 times. The metric was found to outperform all other measures examined by Ulrich and Gotelli [44].
- *Connectance*: The realized proportion of possible links, C = *L* / (*IJ*), where *L* is the realized number of links in a network, *I* is the number of hosts, and *J* is the number of epiphyte species [45].
- *H*_2_ *’*: A network-level measure of specialization based on the deviation of a species’ realized number of interactions and the number expected from each species for a total number of interactions [46] (0 ≤ *H*_2_’ ≤ 1, 0 for no specialization and 1 for perfect specialization).
- Interaction strength asymmetry for host and epiphyte pairs: The interaction between epiphyte and host can be described as an epiphyte individual colonizes a host tree successfully, and vice versa. The positive and negative value depicts the specialization of epiphyte and host, respectively [38].
- *Interaction evenness*: Shannon’s evenness of network interactions [38], which is a metric for detecting if there was potential interaction dominance in the ecological network.
- *Generality* and *Vulnerability*: Concepts borrowed from food web studies. Generality is the number of prey species per predator, and vice versa. Vulnerability is, in our case, the weighted-average number of hosts per epiphytes species and epiphytes species per host [47].

Among these metrics, (i), (ii) and (iii) are nestedness metrics, (iv) measures the amount of fill, (v) and (vi) are specialization metrics and (vii) and (viii) measure the evenness of interaction strength between epiphytes and host trees. Nestedness temperature, NODF, discrepancy score and connectance calculations were based on the binary data matrix and the remainder were all computed based on interaction frequencies (quantitative matrix). The nestedness temperature was calculated using BINMATNEST software, and we used the software's null model 3 to calculate the *p*-value [48]. NODF values for the rows (hosts), columns (epiphytes) and the entire matrix were calculated in Aninhado software [49]. All other indices were calculated in the *bipartite* and *FALCON* package [39, 40] in *R*.

### Ethics statement

This research did not involve human or other animal subjects. For plant collections, we collected the minimum number of specimens required to appropriately voucher field identifications. In most cases, only parts of the plants were collected, although this was not always possible. Permission to conduct research in Mengsong was obtained through a bilateral agreement between Xishuangbanna Tropical Botanical Garden and Xishuangbanna Nature Reserve Bureau at the regular annual meeting between these parties.

## Results

### Field survey

Plots varied in elevation from 1,450 m to 1,786 m a.s.l., plot total tree basal area varied from 22.2 m^2^ ha^-1^ to 45.6m^2^ ha^-1^ and the proportion of trees (≥ 10 cm DBH) colonized by epiphytes varied from 0.56 to 1.0. The number of epiphyte individuals and species recorded per plot varied from 177 to 399 and 21 to 48, respectively. In total, we recorded 77 host trees with 1,756 epiphyte individuals belonging to 103 species; other basic information in the six forest plots surveyed are given in the online supplementary material (S1 Table).

Among the host tree level factors (Table 1), elevation was weakly-or not-correlated to any other variables. Host tree height (*ρ* = 0.7), bark roughness (*ρ* = 0.5), and canopy illumination index (*ρ* = 0.5) were all significantly correlated to host DBH, and host tree height was correlated to stem height (*ρ* = 0.6). To avoid problems of collinearity, tree height, bark roughness, and canopy illumination index were removed from further analysis. Among micro-site parameters, substrate was not significantly correlated with most variables (Spearman's rank correlation, *ρ* < 0.5, Table 1), but was negative correlated with crown position (*ρ* = 0.4, *p* < 0.001). Crown position was significantly correlated to light condition (*ρ* = 0.8) and branch level (*ρ* = 0.8). So, among the micro-site level factors, only crown position and substrate were retained for further analysis.

**Table 1:** Spearman’s rank correlation coefficient of pairwise correlations of Host factors and Microsite factors.

	DBH	HTH	STH	CII	ELE	BAC	BRL	ECP	SUB
**HTH**	0.7***	—	—	—	—	—	—	—	—
**STH**	0.3**	0.6***	—	—	—	—	—	—	—
**CII**	0.5***	0.4***	0.2*	—	—	—	—	—	—
**ELE**	-0.1	-0.2	-0.2*	0	—	—	—	—	—
**BAC**	0.5***	0.3**	0.1	0.3*	-0.1	—	—	—	—
**BRL**	0.6***	0.4**	0.1	0.5***	-0.3*	0.3**	—	—	—
**ECP**	0.6***	0.5***	0.4**	0.5***	-0.4***	0.3**	0.8***	—	—
**SUB**	-0.2	-0.3**	-0.2*	-0.1	0.4***	-0.2	-0.3**	-0.4***	—
**LIT**	0.5***	0.4***	0.3*	0.5***	-0.4***	0.3*	0.7***	0.8***	-0.3**

Note: Abbreviations for Table 1. DBH, Diameter at breast height; HTH, Host tree height; STH, Stem height; CII, Canopy illumination index; ELE, Elevation; BAC, Bark characteristic; BRL, Branch level; ECP, Epiphyte crown position; SUB, Substrate; LIT, Light. Significance level:

*** *p* < 0.001.

** *p* < 0.01.

* *p* < 0.05.

### Diversity

**Fig 3:**
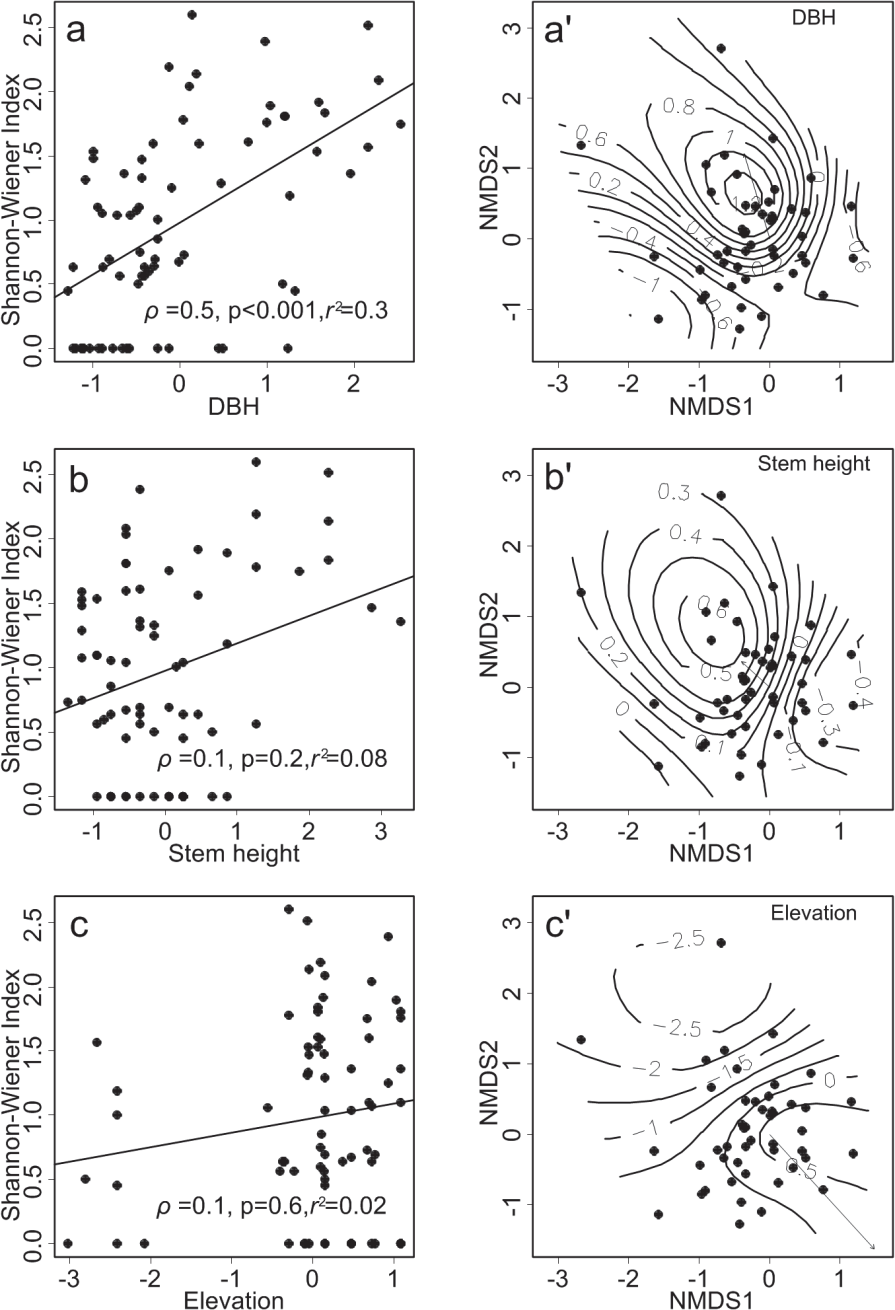
Correlations between epiphyte composition and host tree level environmental factors. Left column: Epiphyte diversity (Shanon-Wiener index) of each host tree against the surveyed environmental factors. Right column: NMDS ordination biplots of the epiphyte community structure. The relationship with different environmental factors is indicated through the contour plots. Ordinations are for visualization only. All statistical tests of factor effects are conducted using *mvabund* (see text for details).

**Fig 4:**
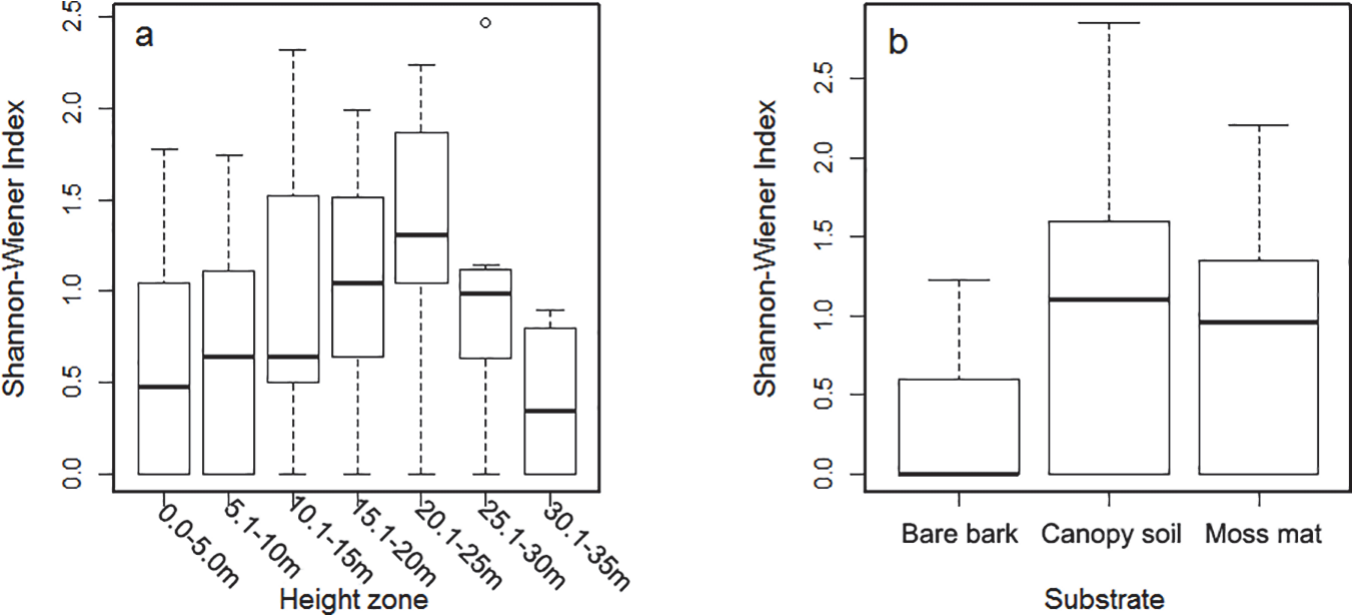
Association between epiphyte diversity and micro-site environmental factors.

Epiphyte diversity (Spearman’s rank correlation) was significantly related with host DBH, but was only weakly or not related to stem height and elevation (Fig 3A–3C, left column). The relationships between epiphyte diversity and micro-site factors was slightly curvilinear (Fig 4A and 4B), indicating a greater epiphyte diversity in mid-canopy positions 10–25 m (Tukey Honest Significant Differences test indicated Shannon-Wiener index was significantly different in height zones 0–5 m vs 15–20 m, *p* < 0.05). Epiphyte diversity varied only slightly between canopy soil and moss mat (Tukey Honest Significant Differences, *p* = 0.4, Fig 4B), but was significantly reduced on bare bark (*p* < 0.001).

### Distribution

After removal of species with IVI of less than 0.01, the site × species matrix had 72 host trees (94% of total hosts) supporting 1,606 epiphyte individuals (91%) belonging to 47 species (46%). The NMDS ordination of epiphytes by hosts, using the Bray-Curtis dissimilarity index, had a final stress of 12% with two dimensions. Stress is a measure of the goodness-of-fit and reflects the adjustment of points required to constrain them to the specified number of axes. A stress of 15% or less is usually considered satisfactory [50]. The fitted contour lines (standardized factors) formed closed circles and irregular curves indicated environmental factors had slight effects on the epiphyte community assembly on each host, and no clear univariate relationships could be detected from the ordination (Fig 3A’–3C’, right column).

### Variance partitioning

To model the effects of the host tree level factors on epiphyte community composition, we used the site × species matrix with 77 host trees and 103 epiphyte species as the dependent variable. The independent variables were the selected host tree level factors. Most of the models with host tree level factors performed better than the null model (Table 2). The best model was epiphyte-host community ~ DBH+STH (stem height) +ELE (elevation) +DBH:STH, which explained 22% of the deviance in the site × species table. Among all single factor models, ~ DBH performed best and explained 6% of the deviance in the site × species table, and ~ DBH + ELE was the best two-factor combination. Thus, the most important host tree level factor in determining epiphyte assembly was host tree size.

**Table 2:** AIC and explained deviance of all possible combinations of host level factors for multivariate models of epiphyte community structure in Bulong Nature Reserve, Mengsong.

Model No.	Variables	Deviance Explained %	AIC
Model 1	STH***+ELE**+DBH:STH*	-63.3	5688.7
Model 2	STH**+DBH:STH	9.1	4783.7
Null	1	0.0	4731.9
Model 3	ELE**	3.8	4730.2
Model 4	DBH:STH**	5.9	4719.0
Model 5	STH***	5.0	4685.3
Model 6	ELE**+DBH:STH**	10.8	4674.6
Model 7	DBH***+STH**+DBH:STH	14.3	4670.8
Model 8	STH**+ELE**	10.5	4647.9
Model 9	DBH***+DBH:STH	10.8	4618.8
**Model 10**	**DBH** ***	**5.9**	**4546.4**
Model 11	DBH***+STH*	11.3	4545.4
Model 12	DBH***+ELE**+DBH:STH	15.7	4545.0
**Model 13**	**DBH** *** **+STH** ** **+ELE** ** **+DBH:STH** *	**22.4**	**4529.1**
**Model 14**	**DBH** *** **+ELE** ***	**11.3**	**4497.6**
**Model 15**	**DBH** *** **+STH** ** **+ELE** *	**18.0**	**4461.2**

Note: Abbreviations for Table 2, please check the notation of Table 1. Bold models were the best performance models. Significance level:

*** *p* < 0.001.

** *p* < 0.01.

* *p* < 0.05.

Among micro-site factors (Table 3), the best model was epiphyte-branch level community~ SUB (substrate) +ECP (crown position) + SUB:ECP, which explained 51% of the deviance of the epiphyte communities across the seven tree zones. Substrate (~ SUB) was the best single factor model, and explained 16% of the deviance in community structure. The best two factor model was ~ ECP + SUB, which explained 30% of the deviance in the site × species table. Crown position (~ ECP) was also important and explained 12% of the deviance of the site × species table.

**Table 3:** AIC and explained deviance of all possible combinations of micro-site factors for multivariate models of epiphyte community structure in Bulong Nature Reserve, Mengsong.

Model No.	Variables	Deviance Explained %	AIC
Null	1	0.0	2321.2
Model 1	ECP:SUB	10.4	2282.1
Model 2	ECP*	11.5	2238.8
Model 3	ECP*+SUB	30.4	2237.9
**Model 4**	**ECP** ^**^ **+ECP:SUB** *	**26.1**	**2208.8**
**Model 5**	**SUB** ^**^	**16.0**	**2185.7**
**Model 6**	**SUB** ^**^ **+ECP+SUB:ECP** *	**50.7**	**2163.9**
**Model 7**	**SUB** ^**^ **+ECP:SUB** *	**37.7**	**2114.4**

Note: Abbreviations for Table 3, please check the notation of Table 1. Bold models were the best performance models. Significance level:

** *p* < 0.01.

* *p* < 0.05.

### Network structure

**Fig 5:**
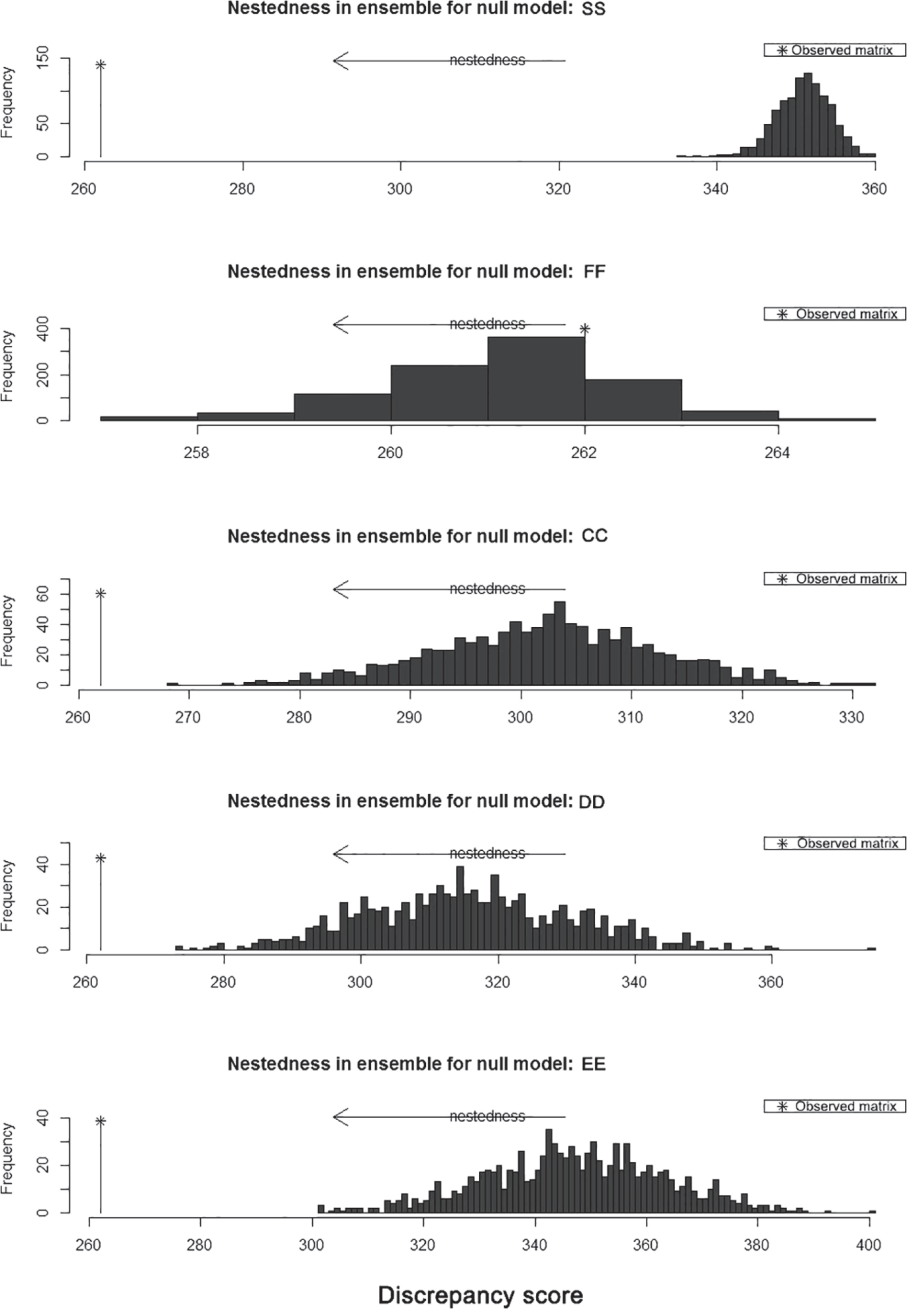
Permutation test of the observed matrix discrepancy score with null models. Null model names were adapted from Beckett et al. [40].

The epiphyte community matrix had a nested temperature (T) of 2.3, and the built-in null models all had significantly higher-average temperatures (Fig 5, T_fisrt_ = 15.2, T_second_ = 10.7, T_third_ = 10.2, all *p* < 0.001). The epiphyte community had a NODF_total_ = 16.4 (N_rows_ = 20.2, N_columns_ = 14.2), which was also significantly higher than the null model (*p* < 0.001; NODF_null total_ = 5.6 and 8.6 for ER, CE null models, respectively). The observed epiphyte host community had a discrepancy score of 262, and the 95% confidential interval of the FF null model was 262 to 266, which suggested a non-nested structure. However, the other four null models produced significantly higher (*p* < 0.001) discrepancy scores indicating a nested structure (Fig 5). Thus according to the results of nestedness measurements, the epiphyte communities supported by each host tree evidenced a highly nested structure, meaning the less diverse epiphyte communities were subsets of more diverse communities (S2 Fig).

**Fig 6:**
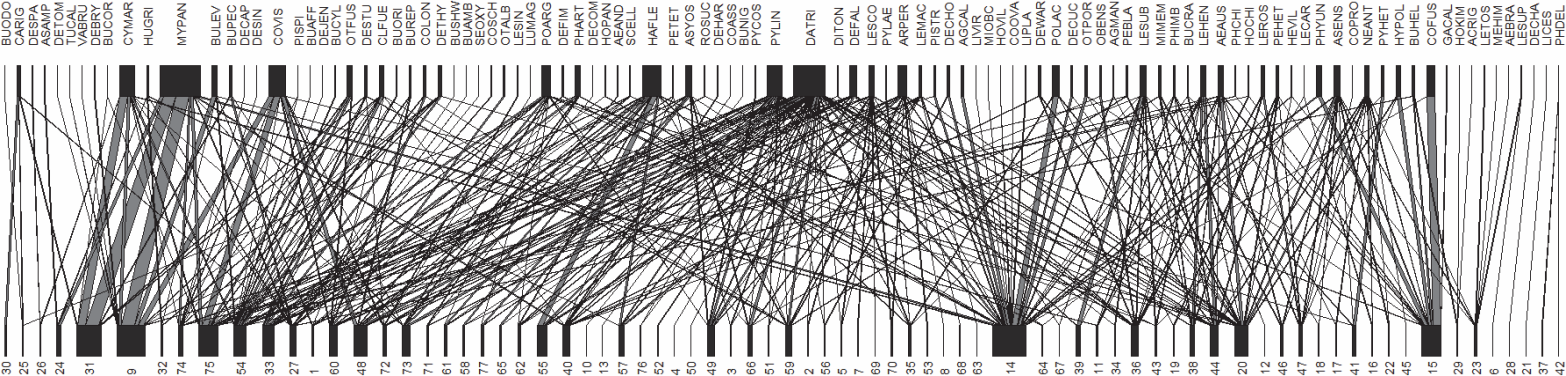
Profiles of the epiphyte-host tree network in Bulong Nature Reserve, Mengsong. Links of between epiphytes and hosts are represented by lines, the widths of the connected rectangles represent the relative frequencies of epiphytes and hosts and the thickness of lines represents the relative frequency of the interactions. Epiphytes are the higher “trophic level”. Epiphyte and host tree names are given in the online electronic supplementary material (S3 and S4 Tables).

The realized proportion of possible links was 5%, indicating the epiphyte-host community was a sparse ecological network. This reflects the low average species richness on each host. The specialization index value *H*_2_’ = 0.5, indicating medium level of specialization between epiphytes and host trees. Interaction strength asymmetry was 0.04; a positive value indicates the higher trophic level species (epiphyte in the present study) were more specialized than host tree [38]. However, as the absolute value was low, this may indicate the specialization asymmetry was slight. Interaction evenness was 0.60 (max = 1), a relative high value, indicating there were few specific interactions that dominated the network[3, 51]. (as depicted by the complicated mesh of black lines of web chart in Fig 6). The interaction evenness of other species tended to be in an even level. An epiphyte species interacted on average with seven (7.2 individual) host individuals (generality), and a host species interacted with eight (7.6 species) epiphyte species (vulnerability), which further underlines the low level of interaction strength asymmetry and specialization.

## Discussion

Studies examining the effects of habitat factors on the structuring of vascular epiphyte communities are particularly scarce in Asia. We surveyed local scale habitat factors and fine scale within host crown level factors affecting the structure of the epiphyte community in a montane forest in SW China. We integrated methods transferred from biogeography and studies of ecological networks to provide a deeper understanding of the structure of epiphyte-host communities. We employed recently developed multivariate generalized linear modeling approaches to partition the variance in epiphyte community composition among hosts and among architectures within host. These methods were specifically developed to deal with the high dimensional data typical of site by species matrices, and the non-normal error structures of species abundance data, and enable one to partition the variance in species composition among independent variables, such as environmental factors. In addition, we employed metrics derived from studies of food webs and pollination networks to characterize epiphyte-host interactions. Together these approaches facilitate understanding of the factors structuring the epiphyte-host community at our site. As a result, we present the most detailed analysis of the ecology of an Asian vascular epiphyte community to date. In general, our results support the findings from other regions. Host size was the most important host level factor determining epiphyte community diversity and composition, while substrate was the most important microsite factor. The epiphyte community was highly nested, indicating that smaller trees contain subsets of the communities found on the largest hosts.

### Effects of factors

Previous studies on the influence of altitudinal gradients on epiphyte community composition have tended to incorporate a substantial gradient, and often included large areas [13, 14, 52, 53]. For instance, a study conducted in Ecuador gathered the complete records of epiphytes from country plant checklists and IUCN red list to assess epiphyte distribution pattern across an elevation range of 0–5,000 m, which was almost the complete altitudinal range for vascular plants in the region, and found that the highest species richness was from 1,000 m to 1,500 m [54]. A mid-elevational peak in epiphyte diversity has been reported from a number of other sites [13, 52]. Our plots were located between 1,450 and 1,786 m and, hence, covered an altitudinal interval of only 336 m. We found that epiphyte community composition varied with elevation, although no obvious pattern was detected. If canopy micro-climatic conditions vary with elevation at our site, which is the most likely explanation of this result, then this suggests that epiphyte species are sorting out along a relatively narrow range of micro-climatic tolerance.

Epiphyte species richness has been shown to increase with host size in many epiphyte studies [5, 10, 55], and host size has also been shown to be an important factor in predicting epiphyte-host network properties [3]. Host size was found to be the most important single factor in structuring the epiphyte community in the present study.

Host size (DBH) is a complex factor that integrates several ecological processes relevant to epiphyte community assembly. It is linked to the exposure time of the host to epiphyte seed rain. It may reflect the time available for the development of an ecological succession within the epiphyte community and a consequent ameliorating of environmental conditions in the crown [8]. Finally, it may reflect the range of environmental conditions from the base of the truck to the tips of the crown, ontogenic change in conditions, such as bark roughness, and the greater available space for epiphytes [10]. Our results are consistent with earlier studies. We found that host size was correlated with other factors that could be important in determining habitat selection in epiphytes, including bark roughness, canopy illumination and tree or stem height. For example, bark roughness could be linked to moisture retention and epiphyte seed germination, as has been discussed in epiphyte studies [1, 3, 11].

Substrate and crown position were important predictors of within crown community structure. Epiphyte abundance and diversity was highest in the middle canopy zones. This supports the idea of a hump-shaped vertical distribution of epiphytes in forest canopy [10, 56, 57]. Humus has been shown to be important in mitigating the strictures of the canopy climatic environment, such as the lack of moisture [16]. However, across hosts we found epiphytes on many different types of substrate. This suggests that epiphyte species may exhibit a range of substrate preferences and our modeling results indicate that qualitative differences in substrate are important in structuring the epiphyte community within host crowns. Overall, the finding that host tree level and within crown micro-site factors influence epiphyte community composition suggests that there are trade-offs among epiphyte ecological strategies with regard to the selection of habitats—such as light, moisture, and nutrient availability [6].

### Community network properties

Through the assessment of indicators for ecological networks, we showed that the epiphyte-host community at our study site was a weakly linked (low filling rate), but highly nested ecological network. The FF null model, which was the only null model not to detect a nested structure in our study, has been found to be too strict and prone to Type II errors [58]. Epiphyte species diversity was also positive correlated with host DBH, which was also the most important factor in determining epiphyte species composition. Together, these results indicate that epiphyte species on smaller host trees were subsets of the species pool on larger host trees. If tree size is correlated with forest age, such a nested structure could potentially arise through whole forest-level changes in the diversity of the epiphyte community [3]. Alternatively, it may arise if more specialist epiphytes can only occur on larger host trees, as suggested by Burns [8].

We also found that the interaction evenness was relatively high and interaction strength asymmetry was low [38], which is consistent with findings elsewhere [3]. This result is perhaps not surprising because trees are essentially only providing structural support for the growth of epiphytes, and the epiphytic habitat in mature forest is highly homogenous at regional scales. Unless the host tree surface has specific devices that reduce the likelihood that epiphytes can colonize, such as alleopathic chemicals in the bark, peeling bark or very smooth bark that may physically shed epiphytes, or harbors protective ants (or other arthropods) that may remove epiphyte seedlings, then host tree characteristics, other than size, may not be so important [31].

Burns’s [8] model of epiphyte community assembly makes several straightforward predictions concerning epiphyte assemblage structure. First, epiphyte species richness should increase with tree size. Second, the epiphyte community structure should be highly nested with the specialist, late-successional species co-occurring with more generalist, early-successional species. Third, host size should be of critical importance in the determining assemblage composition. All three predictions were borne out in our study. Nevertheless, it is also important to point out that host size may represent a number of factors that affect vascular epiphyte abundance and diversity, including the surface area available for colonization, the age of the plant and, thus, the time available for establishment of epiphytes, the light environment because the crowns of larger trees usually occur higher in the canopy, and a greater range of establishment niches. Thus, the importance of a successional process in the assembly of the epiphyte community can only be properly tested through longitudinal studies, which can document the assembly process over time.

## Conclusions

We found that host tree size (DBH) was the most important factor in determining the epiphyte community structure, although the best model explained only 22% of the deviance in epiphyte community structure, suggesting either that other unmeasured factors may be important or that the epiphyte community assembly at the host tree level is highly stochastic [1, 59]. Within hosts, crown position (crown position) and rooting substrate were both important factors in determining epiphyte community composition.

The network parameters indicated the epiphyte-host community at our site was highly nested, the filling rate was low, there was a low level of specialization, and the interaction strengths between epiphytes and hosts was almost equal.

Overall, our results indicate that larger trees harbored a large proportion of the epiphyte community and therefore may be particularly important for the conservation of epiphyte diversity.

## Supporting Information

S1 Fig Comparisons of non-metric multidimensional scaling on the complete epiphyte-host and IVI selected community. A perfect representation could be done in two dimensional NMDS on the complete community (S1 a), however, only several epiphyte species with dissimilarity greater than 3000 units could be shown in an ordiplot (S1 a’). After removal of rare species (56), the remaining 47 epiphyte species could be shown in an ordiplot (S1 b’), with a satisfactory representation (S1 b).
(TIF)

S2 Fig Maximally nestedness packed of the epiphyte host community in Bulong Nature Mengsong. Black rectangles were epiphyte species (103 in total, but repeated), each row represented a host tree (77 in total); the host tree in the left bottom corner represents supporting the least number of epiphyte species, while host tree in the left top corner supporting the largest number. The isocline indicated the perfect nested structure of epiphytes among host trees.
(TIF)

S1 Table Basic information of sampling plots in Bulong Nature Reserve Mengsong. (DOC)

S2 Table Crown illumination index (CII) and definitions. Adapted from Clark and Clark.
(DOC)

S3 Table Checklist of 103 epiphyte species (1756 individuals) in Bulong Nature Reserve Mengsong. (DOC)

S4 Table Checklist of 96 surveyed trees (77 host) in Bulong Nature Reserve Mengsong. (DOCX)
