# Supplementary figures and images for: Structure of the Epiphyte Community in a Tropical Montane Forest in SW China
Source: PLoS One. 2015 Apr 9;10(4):e0122210. doi: 10.1371/journal.pone.0122210 (PMC4391920; doi:10.1371/journal.pone.0122210)

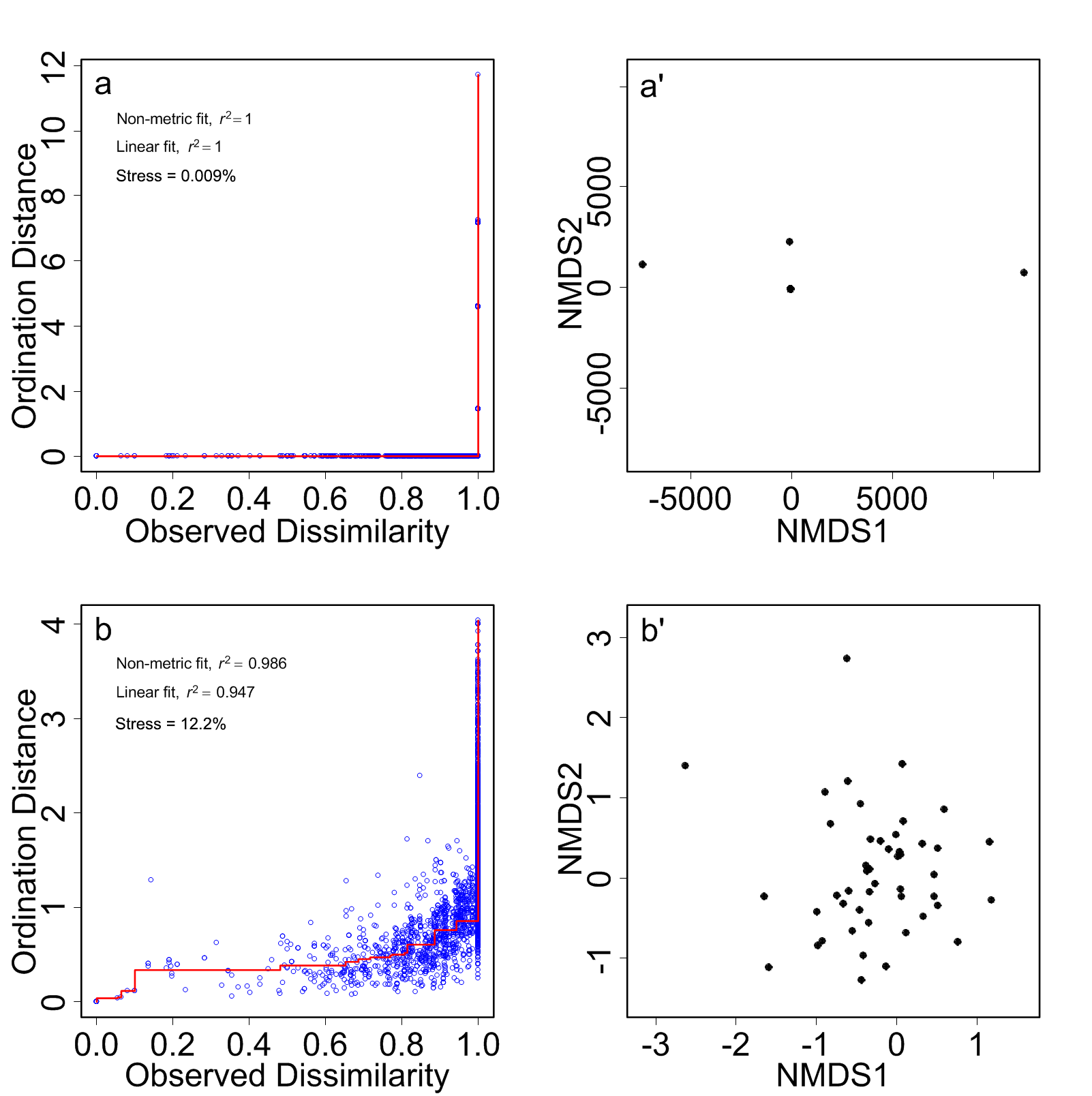

Supplement: S1 Fig — A perfect representation could be done in two dimensional NMDS on the complete community (S1 a), however, only several epiphyte species with dissimilarity greater than 3000 units could be shown in an ordiplot (S1 a’). After removal of rare species (56), the remaining 47 epiphyte species could be shown in an ordiplot (S1 b’), with a satisfactory representation (S1 b). (TIF) [file pone.0122210.s001.tif]

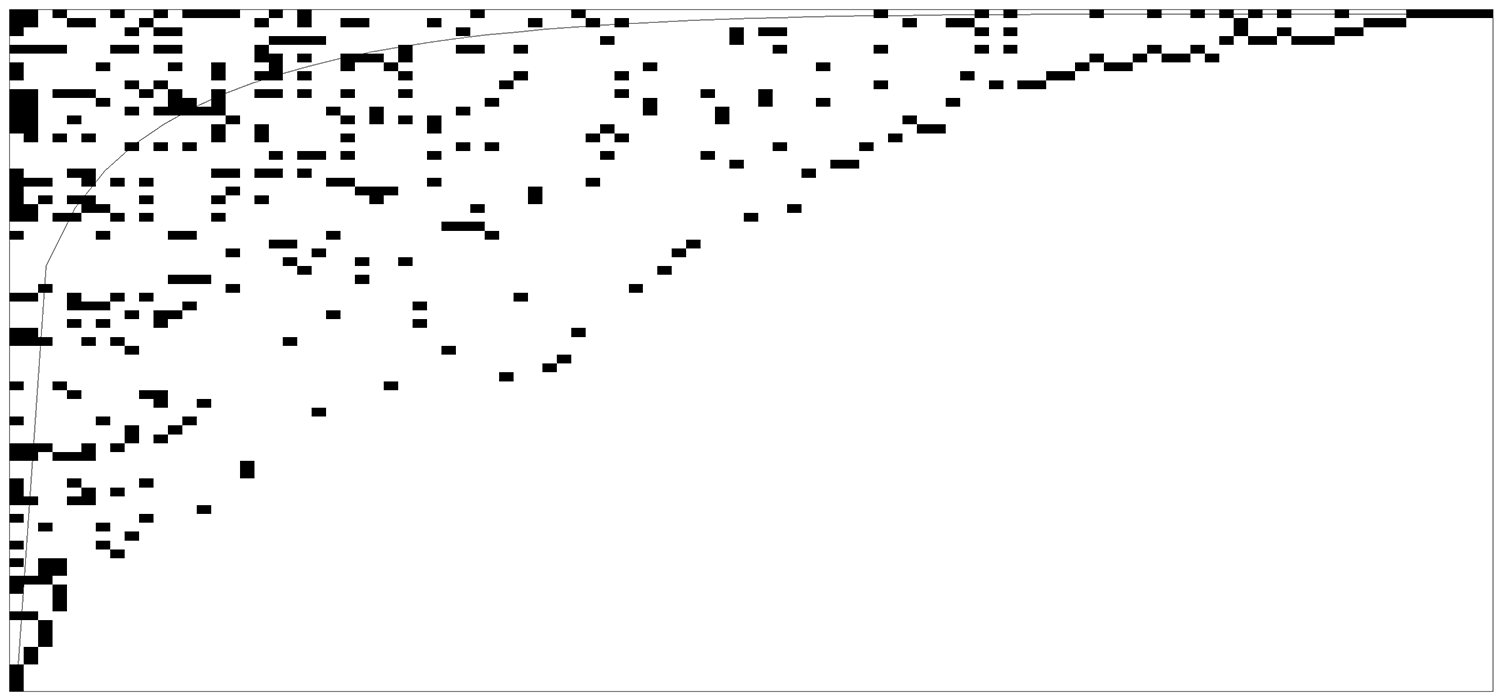

Supplement: S2 Fig — Black rectangles were epiphyte species (103 in total, but repeated), each row represented a host tree (77 in total); the host tree in the left bottom corner represents supporting the least number of epiphyte species, while host tree in the left top corner supporting the largest number. The isocline indicated the perfect nested structure of epiphytes among host trees. (TIF) [file pone.0122210.s002.tif]
