# Supplementary material for: Structure of the Epiphyte Community in a Tropical Montane Forest in SW China
Source: PLoS One. 2015 Apr 9;10(4):e0122210. doi: 10.1371/journal.pone.0122210 (PMC4391920; doi:10.1371/journal.pone.0122210)
Supplement: S1 Table — (DOC) [file pone.0122210.s003.doc]

**Table S1. Basic information of sampling plots in Bulong Nature Reserve Mengsong.**

| **Plot No.** | **Geographic coordinate**  **(E, N)** | **Elevation**  **(m)** | **Vegetation**  **Type** | **Dominate**  **Tree species** | **Host occupancy**  **%** | **Basal area / Plot area**  **(m^2^/ha)** | **No. of Epiphyte Ind. / species**  **(spp.)** | **Mean DBH**  **± SD**  **(cm)** | **Mean Tree height**  **± SD**  **(m)** | **Mean stem height**  **± SD**  **(m)** |
| --- | --- | --- | --- | --- | --- | --- | --- | --- | --- | --- |
| 107 | 100°30'12.77"  21°32'10.30" | 1450 | MEBF | CASMEK,  STYTON | 56.3 | 24.4 | 217/24 | 39.4 ± 20.7 | 14.4 ± 3.1 | 6.5 ± 2.8 |
| 189 | 100°28'27.83"  21°31'6.05" | 1707 | MEBF | CASMEK,  LITMAR | 87.5 | 22.2 | 264/31 | 35.2 ± 21.0 | 20.1 ± 8.8 | 8.2 ± 3.6 |
| 192 | 100°29'20.09"  21°31'5.60" | 1786 | MEBF | CASMEK,  ANNFRA | 100 | 25.6 | 389/30 | 33.5 ± 16.5 | 16.9 ± 5.1 | 7.0 ± 2.7 |
| 214 | 100°28'45.41"  21°30'50.20" | 1673 | TMRF | SYZBRA,  LITBAC | 81.3 | 45.6 | 399/48 | 24.7 ± 17.6 | 18.2 ± 7.8 | 9.9 ± 5.1 |
| 217 | 100°29'37.23"  21°30'49.77" | 1700 | TMRF | ALAKUR,  CRYBRA | 93.8 | 22.2 | 310/36 | 29.1 ± 17.7 | 18.0 ± 7.8 | 9.3 ± 6.8 |
| 240 | 100°30'29.34"  21°30'32.51" | 1747 | TMRF | CALPOL,  CASCAL | 62.5 | 34.1 | 177/21 | 32.3 ± 22.2 | 22.9 ± 10.4 | 10.3 ± 5.7 |
| **General** | **——** | **1677** | **——** | **——** | **80.2** | **29.0** | **293/32** | **32.4 ± 19.4** | **18.4 ± 7.8** | **8.5 ± 4.8** |

Note: MEBF = Monsoon Evergreen Broadleaf Forest, TMRF= Tropical Montane Rain Forest. Dominate trees were selected by the first two importance value in a plot (data in a 1 ha permanent plot was applied, respectively). CASMEK= *Castanopsis mekongensis*, STYTON =*Styrax* *tonkinensis*, LITMAR= *Litsea* *martabanica*, ANNFRA =*Anneslea* *fragrans*, SYZBRA= *Syzygium* *brachythyrsum*, LITBAC= *Lithocarpus* *bacgiangensis*, ALAKUR =*Alangium* *kurzii*, CRYBRA= *Cryptocarya* *brachythyrsa*, CALPOL= *Calophyllum* *polyanthum*, CASCAL= *Castanopsis* *calathiformis*. Host occupancy was calculated by epiphyte occupied trees / total sampled trees (16) in a plot.
