# Supplementary material for: Structure of the Epiphyte Community in a Tropical Montane Forest in SW China
Source: PLoS One. 2015 Apr 9;10(4):e0122210. doi: 10.1371/journal.pone.0122210 (PMC4391920; doi:10.1371/journal.pone.0122210)
Supplement: S2 Table — Adapted from Clark and Clark. (DOC) [file pone.0122210.s004.doc]

**Table S2. Crown illumination index (CII) and definitions. Adapted from Clark and Clark [1]**

| **Index** | **Definition** |
| --- | --- |
| 1 | No direct light, dark area, host tree crown under closed high canopy |
| 2 | Medium lateral light: no direct over-head light and lateral light partially blocked by neighbouring crowns. |
| 3 | Some over-head light, high lateral light (crown lit laterally): < 50% of canopy area exposed to direct over-head light; high lateral light(i.e. neighbouring crowns not obscuring lateral light) |
| 4 | High over-head or full lateral light, high over-head trees with lateral light partially blocked or full lateral light trees with partially over-head light blocked |
| 5 | Crown completely exposed: emergent crown of free-standing |

# Reference

1. Clark DA, Clark DB (1992) Life history diversity of canopy and emergent trees in a neotropical rain forest. Ecol Monogr 62: 315-344.
