# Supplementary material for: Structure of the Epiphyte Community in a Tropical Montane Forest in SW China
Source: PLoS One. 2015 Apr 9;10(4):e0122210. doi: 10.1371/journal.pone.0122210 (PMC4391920; doi:10.1371/journal.pone.0122210)
Supplement: S3 Table — (DOC) [file pone.0122210.s005.doc]

**Table S3. Checklist of 103 epiphyte species (1756 individuals) in Bulong Nature Reserve Mengsong**

| **Family / Epiphyte species** | **Code in**  **Figure 5** | **Individual**  **No.** | **Colonized**  **Tree No.** |
| --- | --- | --- | --- |
| **Araliaceae** | **—** | — | — |
| *Schefflera elliptica* (Blume) Harms | SCELL | 5 | 5 |
| *Tupidanthus calyptratus* J. D. Hooker & Thomson | TUCAL | 1 | 1 |
| **Asclepiadaceae** | **—** | — | — |
| *Dischidia tonkinensis* Costantin | DITON | 2 | 2 |
| *Hoya chinghungensis* (Tsiang & P. T. Li) M. G. Gilbert & P. T. Li & W. D. Stevens | HOCHI | 10 | 4 |
| *Hoya pandurata* Tsiang | HOPAN | 5 | 2 |
| *Hoya villosa* Costantin | HOVIL | 2 | 1 |
| *Micholitzia obcordata* N. E. Brown | MIOBC | 1 | 1 |
| **Aspleniaceae** | **—** | — | — |
| *Asplenium antrophyoides* Christ | NEANT | 24 | 10 |
| *Asplenium ensiforme* Wallich ex Hooker & Greville | ASENS | 35 | 11 |
| *Asplenium yoshinagae* Makino | ASYOS | 37 | 9 |
| **Davalliaceae** | **—** | — | — |
| *Araiostegia perdurans* (Christ) Copeland | ARPER | 48 | 13 |
| *Davallia trichomanoides* Blume | DATRI | 171 | 37 |
| *Humata griffithiana* (Hooker) C. Christensen | HUGRI | 9 | 4 |
| **Ericaceae** | **—** | — | — |
| *Agapetes mannii* Hemsley | AGMAN | 3 | 2 |
| **Gesneriaceae** | **—** | — | — |
| *Aeschynanthus andersonii* C. B. Clarke | AEAND | 1 | 1 |
| *Aeschynanthus austroyunnanensis* W. T. Wang | AEAUS | 28 | 10 |
| *Aeschynanthus bracteatus* Wallich ex A. P. de Candolle | AEBRA | 1 | 1 |
| **Hymenophyllaceae** | **—** | — | — |
| *Hymenophyllum polyanthos* (Swartz) Swartz | HYPOL | 22 | 3 |
| **Melastomataceae** | **—** | — | — |
| *Medinilla himalayana* J. D. Hooker ex Triana | MEHIM | 1 | 1 |
| **Orchidaceae** | **—** | — | — |
| *Acampe rigida* (Buchanan-Hamilton ex Smith) P. F. Hunt | ACRIG | 1 | 1 |
| *Agrostophyllum callosum* H. G. Reichenbach | AGCAL | 12 | 1 |
| *Ascocentrum ampullaceum* (Roxburgh) Schlechter | ASAMP | 4 | 1 |
| *Bulbophyllum affine* Lindley | BUAFF | 1 | 1 |
| *Bulbophyllum ambrosia* (Hance) Schlechter | BUAMB | 1 | 1 |
| *Bulbophyllum corallinum* Tixier & Guillaumin | BUCOR | 2 | 2 |
| *Bulbophyllum crassipes* J. D. Hooker | BUCRA | 6 | 3 |
| *Bulbophyllum cylindraceum* Lindley | BUCYL | 3 | 1 |
| *Bulbophyllum helenae* (Kuntze) J. J. Smith | BUHEL | 12 | 3 |
| *Bulbophyllum levinei* Schlechter | BULEV | 29 | 5 |
| *Bulbophyllum nigrescens* Rolfe | BUNIG | 1 | 1 |
| *Bulbophyllum odoratissimum* (Smith) Lindley | BUODO | 1 | 1 |
| *Bulbophyllum orientale* Seidenfaden | BUORI | 4 | 2 |
| *Bulbophyllum pectinatum* Finet | BUPEC | 14 | 3 |
| *Bulbophyllum reptans* (Lindley) Lindley | BUREP | 11 | 3 |
| *Bulbophyllum shweliense* W. W. Smith | BUSHW | 1 | 1 |
| *Callostylis rigida* Blume | CARIG | 18 | 4 |
| *Cleisostoma fuerstenbergianum* Kraenzlin | CLFUE | 25 | 8 |
| *Coelogyne assamica* Linden & H. G. Reichenbach | COASS | 2 | 1 |
| *Coelogyne fuscescens* Lindley | COFUS | 43 | 2 |
| *Coelogyne longipes* Lindley | COLON | 16 | 3 |
| *Coelogyne ovalis* Lindley | COOVA | 2 | 1 |
| *Coelogyne prolifera* Lindley | COPRO | 1 | 1 |
| *Coelogyne schultesii* S. K. Jain & S. Das | COSCH | 2 | 1 |
| *Coelogyne viscosa* H. G. Reichenbach | COVIS | 92 | 9 |
| *Cylindrolobus marginatus* (Rolfe) S. C. Chen & J. J. Wood | CYMAR | 82 | 9 |
| *Dendrobium brymerianum* H. G. Reichenbach | DEBRY | 5 | 1 |
| *Dendrobium capillipes* H. G. Reichenbach | DECAP | 1 | 1 |
| *Dendrobium chrysanthum* Wallich ex Lindley | DECHA | 1 | 1 |
| *Dendrobium chrysotoxum* Lindley | DECHO | 11 | 2 |
| *Dendrobium compactum* Rolfe ex W. Hackett | DECOM | 1 | 1 |
| *Dendrobium cucullatum* R. Brown | DECUC | 8 | 2 |
| *Dendrobium falconeri* Hooker | DEFAL | 37 | 11 |
| *Dendrobium fimbriatum* Hooker | DEFIM | 6 | 2 |
| *Dendrobium harveyanum* H. G. Reichenbach | DEHAR | 5 | 1 |
| *Dendrobium jenkinsii* Wallich ex Lindley | DEJEN | 1 | 1 |
| *Dendrobium sinominutiflorum* S. C. Chen | DESIN | 1 | 1 |
| *Dendrobium spatella* H. G. Reichenbach | DESPA | 1 | 1 |
| *Dendrobium stuposum* Lindley | DESTU | 17 | 5 |
| *Dendrobium thyrsiflorum* H. G. Reichenbach ex André | DETHY | 21 | 6 |
| *Dendrobium wardianum* Warner | DEWAR | 13 | 3 |
| *Dendrolirium tomentosum* (J. Koenig) S. C. Chen & J. J. Wood | DETOM | 1 | 1 |
| *Gastrochilus calceolaris* (Buchanan-Hamilton ex Smith) D. Don | GACAL | 2 | 1 |
| *Holcoglossum kimballianum* (H. G. Reichenbach) Garay | HOKIM | 2 | 1 |
| *Liparis cespitosa* (Lamarck) Lindley | LICES | 2 | 1 |
| *Liparis platyrachis* J. D. Hooker | LIPLA | 5 | 1 |
| *Liparis viridiflora* (Blume) Lindley | LIVIR | 1 | 1 |
| *Luisia magniflora* Z. H. Tsi & S. C. Chen | LUMAG | 1 | 1 |
| *Mycaranthes pannea* (Lindley) S. C. Chen & J. J. Wood | MYPAN | 215 | 9 |
| *Oberonia ensiformis* (Smith) Lindley | OBENS | 9 | 2 |
| *Otochilus albus* Lindley | OTALB | 8 | 1 |
| *Otochilus fuscus* Lindley | OTFUS | 27 | 4 |
| *Otochilus porrectus* Lindley | OTPOR | 15 | 3 |
| *Phalaenopsis deliciosa* H. G. Reichenbach | PHDEL | 2 | 2 |
| *Pholidota articulata* Lindley | PHART | 27 | 6 |
| *Pholidota chinensis* Lindley | PHCHI | 2 | 2 |
| *Pholidota imbricata* Hooker | PHIMB | 10 | 5 |
| *Pholidota yunnanensis* Rolfe | PHYUN | 29 | 2 |
| *Pinalia spicata* (D. Don) S. C. Chen & J. J. Wood | PISPI | 2 | 2 |
| *Pinalia stricta* (Lindley) Kuntze | PISTR | 8 | 2 |
| *Robiquetia succisa* (Lindley) Seidenfaden & Garay | ROSUC | 5 | 2 |
| *Vanda brunnea* H. G. Reichenbach | VABRU | 1 | 1 |
| **Piperaceae** | **—** | — | — |
| *Peperomia blanda* (Jacquin) Kunth | PEBLA | 10 | 3 |
| *Peperomia tetraphylla* (G. Forster) Hooker & Arnott | PETET | 2 | 2 |
| **Polypodiaceae** | **—** | — | — |
| *Lemmaphyllum carnosum* (Wallich ex J. Smith) C. Presl | LECAR | 2 | 2 |
| *Lemmaphyllum rostratum* (Beddome) Tagawa | LEROS | 17 | 3 |
| *Lepidomicrosorium superficiale* (Blume) Li Wang | LESUP | 4 | 2 |
| *Lepisorus henryi* (Hieronymus ex C. Christensen) Li Wang | LEHEN | 30 | 8 |
| *Lepisorus macrosphaerus* (Baker) Ching | LEMAC | 15 | 9 |
| *Lepisorus scolopendrium* (Buchanan-Hamilton ex Ching) Mehra & Bir | LESCO | 27 | 12 |
| *Lepisorus sinensis* (Christ) Ching | LESIN | 5 | 1 |
| *Lepisorus sublinearis* (Baker ex Takeda) Ching | LESUB | 36 | 9 |
| *Lepisorus tosaensis* (Makino) H. Itô | LETOS | 4 | 1 |
| *Microsorum membranaceum* (D. Don) Ching | MIMEM | 15 | 4 |
| *Polypodiastrum argutum* (Wallich ex Hooker) Ching | POARG | 43 | 13 |
| *Polypodiodes lachnopus* (Wallich ex Hooker) Ching | POLAC | 35 | 4 |
| *Pyrrosia costata* (Wallich ex C. Presl) Tagawa & K. Iwatsuki | PYCOS | 3 | 1 |
| *Pyrrosia heteractis* (Mettenius ex Kuhn) Ching | PYHET | 16 | 2 |
| *Pyrrosia laevis* (J. Smith ex Beddome) Ching | PYLAE | 1 | 1 |
| *Pyrrosia lingua* (Thunberg) Farwell | PYLIN | 78 | 22 |
| *Selliguea oxyloba* (Wallich ex Kunze) Fraser-Jenkins | SEOXY | 2 | 1 |
| **Pteridaceae** | **—** | — | — |
| *Haplopteris flexuosa* (Fée) E. H. Crane | HAFLE | 97 | 13 |
| **Urticaceae** | **—** | — | — |
| *Pellionia heteroloba* Weddell | PEHET | 14 | 5 |
| **Zingiberaceae** | **—** | — | — |
| *Hedychium villosum* Wallich | HEVIL | 5 | 3 |
