# Supplementary material for: Structure of the Epiphyte Community in a Tropical Montane Forest in SW China
Source: PLoS One. 2015 Apr 9;10(4):e0122210. doi: 10.1371/journal.pone.0122210 (PMC4391920; doi:10.1371/journal.pone.0122210)
Supplement: S4 Table — (DOCX) [file pone.0122210.s006.docx]

**Table S4. Checklist of 96 surveyed trees (77 host) in Bulong Nature Reserve Mengsong**

| **No. in Figure 5** | **Family** | **Species** | **Epiphyte**  **individual No.** | **Epiphyte**  **species No.** |
| --- | --- | --- | --- | --- |
| NA | Ebenaceae | *Diospyros kaki* Thunberg | 0 | 0 |
| 1 | Fagaceae | *Castanopsis mekongensis* A. Camus | 7 | 6 |
| NA | Ebenaceae | *Diospyros kaki* Thunberg | 0 | 0 |
| 2 | Theaceae | *Eurya groffii* Merrill | 2 | 2 |
| NA | Theaceae | *Eurya groffii* Merrill | 0 | 0 |
| 3 | Magnoliaceae | *Michelia floribunda* Finet & Gagnepain | 6 | 3 |
| NA | Clusiaceae | *Calophyllum polyanthum* Wallich ex Choisy | 0 | 0 |
| 4 | Clusiaceae | *Calophyllum polyanthum* Wallich ex Choisy | 1 | 1 |
| 5 | Lauraceae | *Cryptocarya brachythyrsa* H. W. Li | 4 | 3 |
| 6 | Lauraceae | *Cryptocarya brachythyrsa* H. W. Li | 1 | 1 |
| NA | Rutaceae | *Tetradium austrosinense* (Handel-Mazzetti) T. G. Hartley | 0 | 0 |
| 7 | Rutaceae | *Tetradium austrosinense* (Handel-Mazzetti) T. G. Hartley | 4 | 1 |
| 8 | Fagaceae | *Lithocarpus truncatus* (King ex J. D. Hooker) Rehder & E. H. Wilson | 1 | 1 |
| NA | Lauraceae | *Cryptocarya brachythyrsa* H. W. Li | 0 | 0 |
| 9 | Theaceae | *Schima wallichii* (Candolle) Korthals | 150 | 12 |
| 10 | Lauraceae | *Phoebe puwenensis* W. C. Cheng | 1 | 1 |
| 11 | Rubiaceae | *Diplospora fruticosa* Hemsley | 10 | 4 |
| 12 | Myrtaceae | *Syzygium brachythyrsum* Merrill & L. M. Perry | 2 | 1 |
| 13 | Euphorbiaceae | *Macaranga henryi* (Pax & K. Hoffmann) Rehder | 1 | 1 |
| 14 | Nyssaceae | *Nyssa javanica* (Blume) Wangerin | 178 | 29 |
| 15 | Myrtaceae | *Syzygium brachythyrsum* Merrill & L. M. Perry | 102 | 14 |
| 16 | Polygalaceae | *Xanthophyllum yunnanense* C. Y. Wu | 2 | 2 |
| 17 | Polygalaceae | *Xanthophyllum yunnanens*e C. Y. Wu | 3 | 2 |
| NA | Euphorbiaceae | *Macaranga indica* Wight | 0 | 0 |
| 18 | Euphorbiaceae | *Baccaurea ramiflora* Loureiro | 3 | 2 |
| 19 | Meliaceae | *Aphanamixis polystachya* (Wallich) R. Parker | 5 | 3 |
| 20 | Magnoliaceae | *Manglietia garrettii* Craib | 73 | 19 |
| 21 | Lauraceae | *Cryptocarya brachythyrsa* H. W. Li | 2 | 1 |
| 22 | Myrtaceae | *Syzygium brachythyrsum* Merrill & L. M. Perry | 4 | 2 |
| 23 | Nyssaceae | *Nyssa javanica* (Blume) Wangerin | 14 | 7 |
| NA | Lauraceae | *Alseodaphne petiolaris* (Meisner) J. D. Hooker | 0 | 0 |
| NA | Actinidiaceae | *Saurauia tristyla* Candolle | 0 | 0 |
| 24 | Theaceae | *Schima wallichii* (Candolle) Korthals | 22 | 3 |
| 25 | Betulaceae | *Betula alnoides* Buchanan-Hamilton ex D. Don | 6 | 3 |
| 26 | Lauraceae | *Machilus tenuipilis* H. W. Li | 4 | 2 |
| 27 | Theaceae | *Schima wallichii* (Candolle) Korthals | 33 | 12 |
| 28 | Theaceae | *Schima wallichii* (Candolle) Korthals | 1 | 1 |
| NA | Lauraceae | *Phoebe puwenensis* W. C. Cheng | 0 | 0 |
| NA | Betulaceae | *Betula alnoides* Buchanan-Hamilton ex D. Don | 0 | 0 |
| NA | Styracaceae | *Styrax tonkinensis* (Pierre) Craib ex Hartwich | 0 | 0 |
| NA | Magnoliaceae | *Manglietia garrettii* Craib | 0 | 0 |
| NA | Betulaceae | *Betula alnoides* Buchanan-Hamilton ex D. Don | 0 | 0 |
| 29 | Fagaceae | *Castanopsis mekongensis* A. Camus | 5 | 3 |
| 30 | Fagaceae | *Castanopsis mekongensis* A. Camus | 8 | 1 |
| 31 | Theaceae | *Schima wallichii* (Candolle) Korthals | 131 | 7 |
| NA | Fagaceae | *Castanopsis echinocarpa* J. D. Hooker & Thomson ex Miquel | 0 | 0 |
| 32 | Theaceae | *Schima wallichii* (Candolle) Korthals | 7 | 3 |
| NA | Fagaceae | *Castanopsis echinocarpa* J. D. Hooker & Thomson ex Miquel | 0 | 0 |
| 33 | Theaceae | *Schima wallichii* (Candolle) Korthals | 60 | 10 |
| 34 | Lauraceae | *Litsea martabanica* (Kurz) J. D. Hooker | 4 | 2 |
| NA | Lauraceae | *Beilschmiedia pergamentacea* C. K. Allen | 0 | 0 |
| 35 | Rubiaceae | *Wendlandia tinctoria* subsp. *intermedia* (F. C. How) W. C. Chen | 14 | 6 |
| 36 | Fagaceae | *Castanopsis mekongensis* A. Camus | 39 | 11 |
| 37 | Araliaceae | *Gymnanthes remota* (Steenis) Esser | 1 | 1 |
| 38 | Alangiaceae | *Alangium kurzii* Craib | 22 | 6 |
| 39 | Alangiaceae | *Alangium kurzii* Craib | 25 | 3 |
| 40 | Lauraceae | *Cinnamomum javanicum* Blume | 37 | 12 |
| 41 | Fagaceae | *Lithocarpus dealbatus* (J. D. Hooker & Thomson ex Miquel) Rehder | 19 | 4 |
| 42 | Polygalaceae | *Xanthophyllum yunnanense* C. Y. Wu | 1 | 1 |
| 43 | Fagaceae | *Lithocarpus dealbatus* (J. D. Hooker & Thomson ex Miquel) Rehder | 12 | 5 |
| 44 | Elaeocarpaceae | *Elaeocarpus sikkimensis* Masters | 43 | 13 |
| 45 | Myrtaceae | *Syzygium brachythyrsum* Merrill & L. M. Perry | 2 | 1 |
| 46 | Lauraceae | *Cryptocarya yunnanensis* H. W. Li | 12 | 4 |
| 47 | Myrtaceae | *Syzygium brachythyrsum* Merrill & L. M. Perry | 19 | 7 |
| 48 | Fagaceae | *Castanopsis mekongensis* A. Camus | 71 | 9 |
| 49 | Araliaceae | *Schefflera bodinieri* (H. Léveillé) Rehder | 39 | 8 |
| 50 | Proteaceae | *Helicia tsaii* W. T. Wang | 2 | 1 |
| 51 | Proteaceae | *Heliciopsis terminalis* (Kurz) Sleumer | 3 | 3 |
| 52 | Fagaceae | *Castanopsis mekongensis* A. Camus | 3 | 1 |
| 53 | Fagaceae | *Castanopsis mekongensis* A. Camus | 1 | 1 |
| 54 | Fagaceae | *Castanopsis mekongensis* A. Camus | 67 | 20 |
| 55 | Fagaceae | *Castanopsis mekongensis* A. Camus | 54 | 5 |
| 56 | Fagaceae | *Castanopsis mekongensis* A. Camus | 3 | 3 |
| 57 | Fagaceae | *Castanopsis mekongensis* A. Camus | 31 | 6 |
| 58 | Fagaceae | *Castanopsis echinocarpa* J. D. Hooker & Thomson ex Miquel | 10 | 3 |
| 59 | Fagaceae | *Castanopsis echinocarpa* J. D. Hooker & Thomson ex Miquel | 33 | 10 |
| 60 | Theaceae | *Schima wallichii* (Candolle) Korthals | 43 | 9 |
| 61 | Fagaceae | *Castanopsis echinocarpa* J. D. Hooker & Thomson ex Miquel | 13 | 4 |
| 62 | Fagaceae | *Castanopsis calathiformis* (Skan) Rehder & E. H. Wilson | 15 | 5 |
| 63 | Theaceae | *Eurya jintungensis* Hu & L. K. Ling | 1 | 1 |
| 64 | Fagaceae | *Castanopsis mekongensis* A. Camus | 4 | 3 |
| 65 | Fagaceae | *Castanopsis mekongensis* A. Camus | 10 | 2 |
| 66 | Fagaceae | *Castanopsis mekongensis* A. Camus | 25 | 6 |
| 67 | Theaceae | *Schima wallichii* (Candolle) Korthals | 2 | 2 |
| 68 | Fagaceae | *Castanopsis mekongensis* A. Camus | 3 | 1 |
| 69 | Fagaceae | *Castanopsis mekongensis* A. Camus | 1 | 1 |
| 70 | Fabaceae | *Albizia chinensis* (Osbeck) Merrill | 6 | 2 |
| NA | Theaceae | *Eurya groffii* Merrill | 0 | 0 |
| NA | Moraceae | *Ficus langkokensis* Drake | 0 | 0 |
| 71 | Fagaceae | *Castanopsis mekongensis* A. Camus | 8 | 3 |
| 72 | Fabaceae | *Albizia chinensis* (Osbeck) Merrill | 16 | 8 |
| 73 | Fagaceae | *Castanopsis mekongensis* A. Camus | 45 | 9 |
| 74 | Fagaceae | *Castanopsis mekongensis* A. Camus | 22 | 11 |
| 75 | Fagaceae | *Castanopsis mekongensis* A. Camus | 104 | 16 |
| 76 | Fabaceae | *Albizia chinensis* (Osbeck) Merrill | 3 | 2 |
| 77 | Myrtaceae | *Decaspermum parviflorum* (Lamarck) A. J. Scott | 15 | 3 |
